# The hepatic sinusoid '*classic and contemporary’*: a report on the 17th international symposium on cells of the hepatic sinusoid (ISCHS)

**DOI:** 10.1186/1755-1536-7-2

**Published:** 2014-01-31

**Authors:** Norifumi Kawada

**Affiliations:** 1 Department of Hepatology, Graduate School of Medicine, Osaka City University, 1-4-3, Asahimachi, Abeno, Osaka 545-8585, Japan

**Keywords:** Stellate cell, Kupffer cell, Sinusoidal endothelial cell, T lymphocyte, Cytokine, Chemokine, Fibrosis, Cancer, Non-alcoholic steatohepatitis, Alcoholic steatohepatitis

## Abstract

The 17th ISCHS took place in Osaka, Japan, on 23 to 25 September 2013. This symposium focuses on an exchange of views on the structure and function of hepatic sinusoidal cells in addition to their roles in clinical pathophysiology.

## Introduction

The 17th International Symposium on Cells of the Hepatic Sinusoid (ISCHS) took place at the Osaka International Convention Center in Nakanoshima, Osaka, Japan, 23 to 25 September 2013. The 17th ISCHS was organized together with the 27th Annual Meeting of Hepatic Sinusoidal Research Japan (HSRJ) and the 20th Annual Meeting of the Japanese Society of the Research of Hepatic Cells as part of Japan Liver Cell Week 2013. In total, 191 scientists from 15 nations attended the 17th ISCHS. The ISCHS originates from the International Kupffer Cell Symposium organized by Professors Eddie Wisse and Dick L Knock on 4 to 7 September 1977 in Noordwijkerhout, The Netherlands. The 17th ISCHS was the third meeting of the International Society for Hepatic Sinusoidal Research (ISHSR), which was founded in 2008. The first ISHSR meeting (15th ISCHS), organized by Professor Hidekazu Tsukamoto, was held in Pasadena, California, USA in 2010, while the second meeting (16th ISCHS), held in Florence, Italy in 2011, was organized by Professor Massimo Pinzani. Professor Kyuichi Tanikawa founded the HSRJ in 1987, and the first HSRJ meeting was held in Kurume, Japan on 14 December 1987. Subsequent annual meetings have been organized at several locations in Japan.

The opening lecture of the 17th ISCHS was given by Kyuichi Tanikawa (Professor Emeritus, Kurume Medical University, Fukuoka, Japan). He discussed his research (representing more than 30 years) and displayed photographs of himself with Professors Eddie Wisse and Hans Popper to show his interaction with the international community. He emphasized the importance of studying the ultrastructure of hepatic sinusoidal cells, particularly with respect to the liver fibrosis and portal hypertension fields.

Following the opening lecture, the scientific sessions of the 17th ISCHS began. The meeting was divided into five thematic sessions: (1) stellate cell and liver fibrosis 1 and 2; (2) inflammation and immunity 1 and 2; (3) cutting edge lecture on hepatic sinusoidal endothelial cells; (4) cancer microenvironment 1 and 2; and (5) alcoholic steatohepatitis (ASH) and non-alcoholic steatohepatitis (NASH) 1 and 2. In addition to oral presentations at the seminars, seven tutorial talks, five invited talks, four keynote lectures, one cutting edge lecture, three communication talks, and 20 selected oral presentations were presented thematically. Of the 44 posters presented, seven were selected for oral presentation in the 'President-selected Short Oral Presentation of Poster’ session.

This meeting was sponsored by the ISHSR and cosponsored by the National Institute on Alcohol Abuse and Alcoholism (NIAAA)/National Institute of Health (NIH), The Southern California Research Center for ALPD & Cirrhosis, Distilled Spirits Council of the United States (DISCUS), and The Uehara Memorial Foundation. This meeting was also supported by industry and the affiliate hospitals of the Department of Hepatology, Osaka City University Medical School (for details, please see Acknowledgements).

### Seminars

In this symposium, two morning seminars, four luncheon seminars, and one evening seminar were planned. These seminars offered information on general and current topics in clinical and basic hepatology, such as hepatitis virus C (HCV), non-alcoholic steatohepatitis (NASH), the diagnosis and treatment of liver fibrosis and cirrhosis, and acute liver failure with a focus on the biology of sinusoidal and other liver cells.

John Peter Iredale (University of Edinburgh, Edinburgh, UK) gave Morning Seminar I, 'Matrix, myofibroblasts and macrophages: players in bidirectional fibrosis’. He emphasized that the hepatic scar is dynamic with respect to both its cellular and matrix components. The resolution of liver fibrosis, even in relatively advanced cirrhosis, occurs cooperatively: activated stellate cell-derived myofibroblasts that abundantly express tissue inhibitor of matrix metalloproteinase (Timp 1) mRNA act in conjunction with macrophages, which are critical in matrix degradation due to their production of matrix metalloproteinases (Mmps) 12 and 13. Restorative macrophages, such as Ly-6C^int^ macrophages, are larger and more complex than pro-fibrotic macrophages. Interaction between macrophages and liver stem cells through TWEAK, Wnt, and Notch should be taken into account.

Takaji Wakita (National Institute of Infectious Disease, Tokyo, Japan) led Morning Seminar II, 'Hepatitis C virus cell culture system and antiviral development’. He is a pioneer in establishing cell culture models of HCV replication and infection. In addition to introducing his analyses of the viral life cycle and his efforts to establish an anti-viral vaccine strategy, he reported that glycyrrhizin, a compound derived from licorice root that is frequently used for treating chronic hepatitis C in Japan, has an inhibitory effect on phospholipase A2 group 1B that prevents the release of infectious HCV particles. Moreover, he noted that HCV entry into stellate cells modulates the activation of these cells.

Four Luncheon Seminars were presented. Massimo Pinzani (University College London-Royal Free Hospital, London, UK) gave the talk 'How our knowledge on liver fibrogenesis can improve the clinical management of chronic liver disease’. He mentioned that advances in the development of non-invasive and repetitive quantification of liver fibrosis can be attributed to progress in serum fibrosis markers and ultrasound-based transient elastography rather than the classical biopsy-based semi-quantitative scoring system of liver fibrosis. Additionally, he stated that in the near future, modern imaging technology will permit the tracking of receptors and signaling molecules involved in the fibrotic process. He also emphasized that cirrhosis is a dynamic clinical stage that should be classified subdivided into several stages to understand its fundamental reversibility and to develop therapeutic strategies.

Satoshi Mochida (Saitama Medical University, Saitama, Japan) gave the talk 'Acute liver failure in Japan-definition, classification and prediction of the outcome’. Acute liver failure is caused by massive hepatocyte necrosis. This condition occurs in patients infected with hepatitis virus A or B, autoimmune hepatitis or certain other diseases, and is accompanied by blood coagulation disequilibrium in the hepatic sinusoid. Although the pathogenesis of acute liver failure remains to be clarified, the Intractable Hepato-Biliary Disease Study Group of Japan reported diagnostic criteria for this disease and a scoring system to predict patient outcome in 2011. Therapeutic strategies other than liver transplantation for this rare but lethal liver disease should be established based on the outcome of cell-based mechanical analyses.

Gyongyi Szabo (University of Massachusetts Medical School, Worcester, MA, USA) presented 'Inflammation and danger signals in alcoholic and non-alcoholic fatty liver disease’. Her lecture on the pathogenesis of ALD and NAFLD focused on endogenous and exogenous danger signals. Innate signaling molecules and pathways, such as toll-like receptors (Tlrs) and inflammasomes, contribute to inflammation in ALD and NAFLD. Activation of the Nlr family, pyrin domain containing 3 (Nlrp3) inflammasome, and caspase-1/interleukin 1β (Il-1β) is involved in the pathogenesis of ALD.

Kazuaki Chayama (Hiroshima University, Hiroshima, Japan) presented 'Studies on hepatitis viruses using human hepatocyte chimeric mice’. He utilized human hepatocyte chimeric mice to study HBV and HCV virology. Human hepatocytes were transplanted into albumin enhancer/promoter-driven urokinase plasminogen activator/severe combined immunodeficient (uPA/SCID) mice to generate the first human hepatocyte chimeric mice. This innovative mouse model has utility for both anti-viral drug screening and analyses of viral dynamics. He commented on the current status of their group’s studies using this mouse model to explore the life cycles of HBV and HCV and to develop new anti-viral therapeutic strategies.

Takehiro Okabayashi (Kochi Health Science Center, Kochi, Japan) gave the talk 'Perioperative nutritional management using branched chain amino acid (BCAA) in hepatic resection patients’ at Evening Seminar I. BCAA supplements are used therapeutically to improve the nutritional status and quality of life of patients with end-stage liver disease and cirrhosis. Recent studies have revealed that BCAA can prevent cirrhosis-related complications and prolong the survival of cirrhotic patients by suppressing the occurrence of hepatocellular carcinoma (HCC); however, a detailed molecular mechanism is lacking. In this lecture, he showed novel results indicating that BCAA supplementation supports hepatocyte proliferation by stimulating the migration of extrahepatic stem cells from the bone marrow into the site of liver damage.

### Stellate cells and fibrosis

This session was composed of three tutorial talks, four invited talks, and five oral presentations selected from submitted abstracts. Tutorial Talk 1 was given by Kenjiro Wake (Professor Emeritus, Tokyo Medical and Dental University/Minophagen Pharmaceutical Co. Ltd., Tokyo, Japan) who contributed to the 're’ discovery and confirmation of the presence of hepatic stellate cells in the space of Disse. Using microscopy and electron microscopy technologies, he determined that these stellate cells contain lipid droplets of vitamin A within their cytoplasms. Furthermore, the 3D structure of these stellate cells, revealed both by Golgi’s silver staining method and by a cell culture model, suggested that they are liver-specific pericytes that regulate sinusoidal microcirculation via their contractile ability. He also described the heterogeneity of these stellate cells and their interactions with neighboring hepatocytes and endothelial cells.

Kinji Asahina (University of Southern California, Los Angeles, CA, USA) gave a talk on the role of mesothelial cells in liver fibrosis. Conditional cell lineage tracing revealed that mesothelial cells are able to transdifferentiate into hepatic stellate cells, fibroblasts, and smooth muscle cells during liver development. Mesothelial-mesenchymal transition occurs in response to transforming growth factor β (Tgf-β) stimulation and gives rise to hepatic stellate cells and myofibroblasts, thereby suggesting that these cells contribute to the capsular fibrosis of the chronically damaged liver. Based on this report, myofibroblasts may be derived from at least three different sources in the liver: hepatic stellate cells, portal myofibroblasts, and mesothelial cells.

Chantal Housset (Saint-Antonie Research Center, Paris, France) discussed myofibroblast sub-populations in the liver. She mentioned that myofibroblasts in the liver originate from hepatic stellate cells and portal/periportal myofibroblasts. However, there are some differences between the two cell types; portal myofibroblasts play dominant roles in scar formation, while hepatic stellate cell-derived myofibroblasts are important in the healing process. These two cell populations have different functions during hepatic vascular remodeling.

Eva Moran’s (University of Barcelona, Barcelona, Spain) presentation on cell-specific *peroxisome proliferator-activated receptor γ* (*Pparγ*) deficiency using the Cre-LoxP gene targeting system revealed anti-inflammatory and anti-fibrogenic properties for this nuclear receptor in non-parenchymal liver cells. Mice with macrophage-specific deletion of *Pparγ* (*Pparγ*f/-LysM-Cre) showed marked necroinflammatory injury and lipid peroxidation, an augmented fibrogenic response to chronic carbon tetrachloride (CCl_4_) intoxication, activation of caspase 3 and caspase 3/7, and increased expression of cyclooxygenase 2 (Cox-2), tumor necrosis factor α (Tnfα), and Il-1. Identical results were obtained by the specific deletion of *Pparγ* in stellate cells (*Pparγ*f/-aP2-Cre). She concluded that Ppar*γ* expression in non-parenchymal liver cells is protective against inflammation and fibrosis in liver injury.

Takeshi Saito (University of Southern California, Los Angeles, CA, USA) gave a talk on the retinoid regulation of hepatic antiviral innate immunity. He showed that alcohol dehydrogenase (Adh) expression in a cell line enhances retinoic acid (RA) production in hepatocytes, resulting in the enhanced expression of interferon-stimulated gene (Isg) and the potentiation of the antiviral action of interferon (Ifn) in the induction of Isg. Thus, vitamin A-rich, quiescent stellate cells supply retinols to hepatocytes, thereby facilitating their antiviral innate immunity.

Yoon Seok Noh (University of California, San Diego, La Jolla, CA, USA) showed that the Tlr7-type 1 Ifn signaling axis inhibits cholestasis- and hepatotoxin-induced liver fibrosis through the induction of Il-1 receptor antagonists. Using both KO mice and a cell culture model, he demonstrated that Tlr7 signaling induces dendritic cells to produce type I Ifn (α and β). Secreted type I Ifn triggers the production of an Il-1 receptor antagonist by Kupffer cells. Finally, this Il-1 receptor antagonist regulates the bile duct ligation-induced liver fibrosis process. These results elucidate the detailed molecular mechanism by which clinically utilized type I Ifn represses fibrosis.

Hidekazu Tsukamoto (Keck School of Medicine of the University of South California, Los Angeles, CA, USA) gave Tutorial Talk 2 in this session. He commented on facts and wonders of hepatic stellate cells. He emphasized that *Pparγ* is a key gene regulating the differentiation or quiescence of stellate cells. Its epigenetic repression, mediated by morphogens such as Wnt, Shh, Necdin, and Dlk1, induces a myofibroblastic cell fate. He also discussed the role of stellate cells in hepatic oncogenesis.

Sophie Lotersztajn (Hospital Henri Mondor, Creteil, France) gave Tutorial Talk 3 on cannabinoid receptors as antifibrogenic targets during chronic liver disease. CB1 receptor antagonists display beneficial effects on lipid metabolism and have anti-fibrotic properties. The CB2 receptor is a promising antifibrotic target that has indirect anti-inflammatory effects on Kupffer cells and Th17 lymphocytes.

Yasuko Iwakiri (Yale University School of Medicine, New Haven, CT, USA) gave Invited Talk 3. The talk focused on Nogo-B (Reticulon 4B), a novel regulator of liver fibrosis and portal hypertension. Nogo-B belongs to the reticulon family of proteins and is present in the endoplasmic reticulum (ER), where it regulates ER structure and protein trafficking. Using *Nogo-B* deficient mice, she demonstrated that Nogo-B attenuates liver fibrosis by disturbing the Tgf-β/Smad2 pathway and inducing apoptosis of activated stellate cells. Nogo-B is also involved in the development of portal hypertension.

Klaas Poelstra (University of Groningen, Groningen, The Netherlands) gave Invited Talk 4. He discussed maneuvering cytokines to modulate fibrogenesis. When truncated, Ifn *γ* acts as a mimetic by binding the platelet-derive growth factor (Pdgf) receptor instead of its own receptor, thereby inducing significant antifibrotic effects. In contrast, pegylated Il-10, which has potent antifibrotic effects, exhibits significant pro-fibrotic effects when delivered to the Pdgf receptor *in vivo*. Thus, the cell-specific delivery of cytokines to hepatic sinusoidal cells modulates disease activity.

Yutaka Furutani (Riken Center for Life Science Technologies, Saitama, Japan) gave a selected oral presentation on reduced angiogenesis and fibrosis in the liver of *transglutaminase 2*-deficient mice after bile duct ligation. *Transglutaminase 2*-deficient mice injected with VEGF display a 50% reduction in angiogenesis and liver fibrosis compared to wild-type mice, indicating that transglutaminase 2 is involved in Tgf-β activation. The latent form of Tgf-β is produced by neovessels.

Zhimin Zhao (Shanghai University of Traditional Chinese Medicine, Shanghai, China) gave a talk on the inhibition of liver fibrosis and angiogenesis *in vivo* and *in vitro* by levistilide A. Levistilide A is derived from the herb *Angelica Sinensis* (Danggui) and has been used to treat liver disease in China. This compound caused reduced expression of von Willebrand factor and attenuated CCl_4_-induced liver fibrosis and sinusoidal capillarization.

### Communications on 'Fibrosis in extrahepatic organs’

Communications on 'Fibrosis in extrahepatic organs’ was first programmed in ISCHS. It has been well established that fibroblast-like cells similar to hepatic stellate cells and myofibroblasts reside not only in the liver but also in the pancreas, kidney, lung, gut, spleen, and other visceral organs. Recent studies have demonstrated that these stellate cells in extrahepatic organs, some of which are specialized to store vitamin A, play a myriad of roles in the development of fibrosis within individual organs. In this session, the molecular and cellular basis of fibrosis in the lung, kidney, and pancreas were discussed in this context.

Thomas A Wynn (National Institute of Allergy and Infectious Disease, NIH, Bethesda, MD, USA) gave a talk on the roles of Tgf-β1, Il-13, and inflammatory mediators in mouse models of pulmonary fibrosis. He mentioned that pulmonary fibrosis induced by chronic allergen exposure is Il-13 dependent, while bleomycin-induced fibrosis is mediated by Il-17A produced by CD4^+^ T cells and *γ*δ^+^T cells. Pulmonary fibrosis is suppressed by Il-10 and augmented by Ifn *γ* and Il-12/23p40. Thus, he concluded that both Il-13 and Il-17A are important mediators of pulmonary fibrosis.

Motoko Yanagita (Kyoto University, Kyoto, Japan) gave a talk on the origin and function of scar-forming cells in the kidney. Although epithelial-mesenchymal transition (EMT) takes part in some myofibroblasts in the fibrotic kidney, the trans-differentiation of erythropoietin-expressing, neural crest-derived fibroblasts into Pdgf β- and αSma-positive myofibroblasts is dominantly involved. Selective estrogen receptor modulators such as tamoxifen can reverse this process. She also emphasized that epithelial-fibroblast interactions trigger fibroblast dysfunction during kidney injury.

Astushi Masamune (Tohoku University, Sendai, Japan) gave a talk on the role of pancreatic stellate cells in pancreatic fibrosis. There are vitamin A-positive, periacinar, stellate-shaped cells in both the human and rodent pancreas. Like hepatic stellate cells, pancreatic stellate cells undergo trans-differentiation from a quiescent phenotype to an αSma-positive and ECM-producing activated phenotype. In addition to exhibiting profibroblastic characteristics, pancreatic stellate cells are involved in insulin secretion by β-cells and in the resistance of cancer cells to chemotherapy and radiotherapy.

### Inflammation and immunity

This session was composed of two tutorial talks and five oral presentations selected from submitted abstracts. Laura Nagy (Cleveland Clinic, Cleveland, OH, USA) gave Tutorial Talk 4. She discussed the role of complement in alcoholic and non-alcoholic liver disease. She described data from several types of KO mice to demonstrate that ethanol feeding induces the activation of the complement cascade, which, in turn, stimulates sinusoidal Kupffer cells to produce Tnf-α, thereby triggering hepatocyte damage. C1q and the classical complement pathway are critical mediators of both ASH and NAFLD.

Following the tutorial talk, Maria Lauda Tomasi (Keck School of Medicine of the University of South California, Los Angeles, CA, USA) gave a talk on the molecular mechanism of lipopolysaccharide (LPS)-mediated inhibition of glutathione synthesis in murine macrophages. LPS lowers the expression of glutamate-cysteine ligase by mediating decreased sumoylation of nuclear factor erythroid 2-related factor 2 (Nrf2) and MafG. LPS treatment reduced the co-occupancy of Nrf2 and MafG on the same promoter region containing the antioxidant response element. This report showed that the LPS-mediated downregulation of protein sumoylation is involved in the expression of antioxidant defense system genes.

Silvia Affo (University of Barcelona, Barcelona, Spain) gave a talk on the role of the chemokine receptor (Ccr) 6 in the pathogenesis of chronic and acute-on-chronic liver disease. *Ccr6*^-/-^ mice showed augmented expression of both pro-inflammatory (monocyte chemotactic protein (Mcp)-1, Icam-1, and Il-6) and pro-fibrogenic (collagen 1a1 (Col1a1) and Tgf-β) mRNAs in response to liver injury. She also observed the increased recruitment of an altered CD4^+^ and CD4^+^Foxp3^+^ dendritic cell population that led to the exacerbation of liver inflammation and fibrogenic reactions in *Ccr6*^-/-^ mice.

Hartmut Jaeschke (University of Kansas Medical Center, Kansas City, KS, USA) gave the second tutorial talk in this session (Tutorial Talk 5). Acetaminophen (APAP) is one of the most commonly used drugs worldwide and occasionally induces fatal acute liver failure (400–500 deaths/year in the US). An intermediate metabolite of APAP, NAPQI, induces necrosis in hepatocytes; however, the innate immune response to this process remains to be clarified. He demonstrated that APAP-induced liver damage is not augmented by exogenous Il-1β and occurs in *Il-1 receptor*-, *caspase 1*-, and *Nalp3*-deficient mice, indicating that the activation of the Nalp3 inflammasome has no relevant impact on APAP hepatotoxicity in mice. Infiltrating macrophages are critical for the recovery phase of APAP-induced liver injury.

Yoshihiro Kamada (Osaka University, Osaka, Japan) gave a talk on the effect of N-acetylglucosaminyltransferase V (GnT-V) on the progression of chronic hepatitis with steatosis. GnT-V catalyzes beta 1–6 branching in asparagine-linked oligosaccharides. The expression of a *GnT-V* transgene under control of the beta-actin promoter prevents inflammatory and fibrogenic reactions in mice fed a high-fat, high-cholesterol diet. Although hepatic stellate cells isolated from *GnT-V* transgenic mice expressed high levels of Tgf-β1 mRNA, they expressed lower levels of collagen 1a1 compared to stellate cells isolated from wild-type mice. This discrepancy can be explained by the overexpression of cyclooxygenase-2 and the resultant overproduction of prostaglandin E2, which negatively regulates collagen 1a1 mRNA expression in stellate cells isolated from *GnT-V* transgenic mice.

Ayako Mabuchi (University of Otago, Dunedin, New Zealand) gave a talk on the role of Tlr4 in stellate cell activation and hepatocyte proliferation during liver regeneration. Partial hepatectomies (70%) were performed in wild-type mice, *Tlr4* deficient mice, and mice treated with MTS510 (a rat anti-mouse Tlr4/Md2 monoclonal antibody). After hepatectomy, DNA synthesis in hepatocytes was comparable among the groups, while an increase in ALT was limited in to *Tlr4*-deficient mice and mice treated with MTS510. Three days after PH, the livers of *Tlr4*-deficient mice showed an incomplete regenerative process: NF-κB activation was not present, and αSma-positive activated stellate cells were undetectable. Accordingly, she concluded that stellate cell activation, but not hepatocyte proliferation, is Tlr-dependent during liver regeneration.

Andrej Khandoga (University of Munich, Munich, Germany) gave a talk on the interactions between hepatic stellate cells and T cells during hepatic ischemia-reperfusion (I/R) injury in mice. T cell-hepatic stellate cell interactions were visualized *in vivo* after the infusion of fluorescein-labeled CD4^+^ T cells into Cx3CR1 mice harboring GFP-labeled stellate cells. The results demonstrated that co-localization of CD4^+^ T cells and stellate cells was evident after I/R and was attenuated by JWH133, a CB-2 agonist. ACPA, a CB-1 agonist, had no effect. In addition, JWH133 treatment significantly attenuated I/R-dependent hepatocyte damage, indicating that a selective depletion/deactivation of stellate cells reduces T cell-dependent I/R injury.

### Cutting edge lecture on sinusoidal endothelial cells

Laurie DeLeve (Keck School of the University of Southern California, Los Angeles, CA, USA) reviewed the topic of liver sinusoidal endothelial cells in liver regeneration. Liver sinusoidal cell progenitor cells (SPCs) are composed of resident (intrahepatic) SPCs and bone marrow SPCs (BM SPCs). BM SPCs are required for the repair of liver sinusoidal endothelial cells (LSECs) after both toxic injury and partial hepatectomy and accumulate in the liver in response to hepatic Vegf. Stromal cell-derived factor 1 acts downstream of Vegf to regulate BM SPC recruitment and engraftment. BM SPCs are the major source of hepatocyte growth factor (Hgf) and promote hepatocyte proliferation, while resident SPCs are presumed to play a role in normal LSEC turnover.

### Cancer microenvironment

This session was composed of three keynote lectures, one invited talk, and four oral presentations selected from the submitted abstracts. In previous ISCHS symposia, the anatomical and histological features of hepatic sinusoidal cells and their involvement in the fibrotic reaction, local inflammation and immunity, and sinusoidal microcirculation have been principally discussed. Recently, more clinically relevant roles of sinusoidal cells have also been discussed. The session entitled 'Cancer Microenvironment’ outlined current trends.

Ekihiko Seki (University of California, San Diego, La Jolla, CA, USA) gave keynote lecture 1. He is interested in liver fibrosis and liver cancer development. Mice deficient in *transforming growth factor (Tgf)-β-activated kinase 1* (*Tak1*) in hepatocytes (*Tak1*^*∆Hep*^) showed spontaneous development of liver cancer accompanied with hepatocyte death (increased ALT) and marked fibrosis. *Tak*^*∆Hep*^ livers showed marked activation of the Smad 2/3 pathway. Furthermore, the deletion of both *Tak1* and *Tgf*β*r2* in hepatocytes obviously reduced the number of tumors developed and the extent of fibrosis, indicating that *Tgf*β-signaling is a prerequisite for liver cancer development in *Tak*^*∆Hep*^ mice. Further analyses revealed the contribution of Smad 4 in hepatocytes during cancer development in *Tak*^*∆Hep*^ mice. In the last part of his talk, he further emphasized the importance of Tgf-β signaling in hepatocyte cell death and lipid accumulation in a mouse model of NASH.

Shin Maeda (Yokohama City University, Yokohama, Japan) gave keynote lecture 2. E-cadherin (Cdh) is an important cell-cell adhesion molecule that generates adherence junctions; however, its role in tumor development remains controversial. Both hepatocyte-specific Cdh-deficient mice (*Cdh*^*∆L*^) and bile duct epithelial-specific *Cdh*-deficient mice developed fibro-inflammatory lesions around bile ducts without displaying obvious derangement in bile duct epithelial cell morphology. *Cdh*^*∆L*^ mice exhibited augmented expression of Sox9, Cd44, and K19 around bile ducts and developed spontaneous liver tumors in some cases. The Kras and ERK signaling cascades mediated this process. Furthermore, he explained that the knockdown of Cdh in Hep3B cancer cells upregulated EMT and the expression of stem cell markers such as Cd44. He concluded that E-cadherin is a true tumor suppressor in the liver.

Eiji Hara (Japanese Foundation for Cancer Research, Tokyo, Japan) gave keynote lecture 3. Obesity is a risk factor for developing cancer, although the mechanism connecting these two pathologic conditions remains undetermined. He reported that the involvement of cellular senescence and senescence-associated secretary phenotypes (SASPs) in tumor formation is caused by obesity, using mice fed a high-fat diet (HFD) and given DMBA, a compound that induces H-ras mutations. Interestingly, he determined that senescent cells in HFD-fed mouse livers are hepatic stellate cells expressing p21, p16, *γ*H2AX, and αSma. The depletion of senescent hepatic stellate cells using si-heat shock protein 47 decreased tumor development. Taken together, these data demonstrate that HFD-induced SASPs in stellate cells are a prerequisite for cancer development in this model. Furthermore, he demonstrated that deoxycholic acid (DCA), a gut bacterial metabolite, contributes to the induction of senescence in stellate cells. The gram-positive bacteria *Clostridium Cluster XI* principally produce DCA in the gut.

Fernando Vidal-Vanaclocha (CEU-San Pablo University, Madrid, Spain) gave Invited talk 5. This talk described the mechanism of colon cancer cell metastasis to the liver. Colon cancer induces the production of factors required for 'liver prometastatic reaction’ by creating a special microenvironment supporting the colonization of hepatic sinusoidal cells by metastatic cancer cells. Il-1β, Il-1β converting enzyme, or Ddr2 deficiency have a similar microenvironment that promotes liver prometastatic reaction. He further described microenvironment-related genes whose expression levels were regulated by hepatocytes, hepatic myofibroblasts or both, as well as genes representative of the liver prometastatic reaction.

Hayato Hikita (Osaka University, Osaka, Japan) reported that liver carcinogenesis is induced by continuous apoptosis and oxidative stress in hepatocytes. Continuous hepatocyte apoptosis was observed in *Bcl-xL* or *Mcl-1*-deficient mice. More than 88% of these mice develop liver cancer within 1.5 years compared to 0% of wild-type mice. Deep sequencing analyses revealed that mutations in *p53* and β*-catenin* were not involved. However, the methylation rates of *runx3* in both cancerous and non-cancerous areas were significantly higher in KO mice compared to wild-type mice. Furthermore, hepatocyte apoptosis was found to induce oxidative stress accompanied by the induction of the anti-oxidant enzymes, nqo1 and ho1. Although N-acetylcysteine treatment has no effect on inflammation and fibrosis in KO mice, this treatment markedly decreased the incidence of liver cancer.

Thuy T Le (Osaka City University, Osaka, Japan) presented a study on liver cancer development in *cytoglobin*-deficient mice treated with diethylnitrosamine (DEN) or choline-deficient amino acid-defined (CDAA) diet. Mice in both groups exhibited a high incidence of tumor development accompanied by significant fibrosis and augmented oxidative stress. Of note, hepatic stellate cells isolated from *cytoglobin*-deficient mice were primed and secreted multiple cytokines involved in the fibro-inflammatory reaction in the liver. Because cytoglobin is uniquely expressed in stellate cells, these findings indicate the direct involvement of stellate cells in cancer development in the mouse liver.

Hitoshi Yoshiji (Nara Medical University, Nara, Japan) gave a talk on the effects of combined treatment of sorafenib, a multi kinase inhibitor, and angiotensin II receptor blocker (ARB) on the development of preneoplastic lesions in CDAA-treated rat livers. Both agents are known to have strong anti-angiogenic activity. Sorafenib (5 mg/kg/day) showed marked inhibition of the development of GST-P-positive neoplastic lesions. However, a combined treatment of ARB, losartan (30 mg/kg/day) and a half dose of sorafenib (2.5 mg/kg/day) exhibited equivalent anti-tumor effects, along with hepatic neovascularization and Vegf expression. Accordingly, he concluded that this regimen using a lower dose of sorafenib might provide a new strategy for treating HCC caused by NASH.

Takuji Torimura (Kurume University School of Medicine, Fukuoka, Japan) presented the final talk of this session. He reported anti-tumor effects of aflibercept, a soluble decoy receptor made by fusing VEGF receptors (VEGFR)-1 and -2 that has the ability to bind both VEGF and PlGF. Aflibercept suppressed the phosphorylation of VEGFR-1 and -2 in human umbilical cord cells and hepatoma cells, resulting in inhibited cell proliferation. It also inhibited the differentiation of bone marrow (BM) cells into endothelial progenitor cells and the migration of BM cells to tumor tissues. In tumor-bearing mice, aflibercept reduced the microvascular density and growth of tumor tissues without serious adverse events.

### NASH and ASH

This session was composed of two tutorial talks, one keynote lecture, and six oral presentations selected from submitted abstracts. Fabio Marra (University of Florence, Florence, Italy) gave the first tutorial talk in this session on the fibrogenic mechanisms in ASH and NASH. Lipotoxicity induces hepatocyte dysfunction via apoptosis, ER stress, oxidative stress, and so on, thereby leading to stellate cell activation and fibrosis. Ccl2 produced in adipose tissue plays a role in hepatocyte steatosis, recruitment of inflammatory cells into the liver and oxidative stress. Berberine (BRB), a plant alkaloid with a long history of medical use, ameliorates MCD-induced steatohepatitis and causes reduced expression of Il-1β, Il-18, and pannexin-1. In addition, he discussed studies that used siRNA, the small molecule XMD8-92, and human cancer tissues to determine the role of ERK5 in HCC growth.

Ariel Feldstein (University of California, San Diego, La Jolla, CA, USA) gave keynote lecture 4 on NAFLD, NASH and cirrhosis. He reported that MCD diet- and CDAA diet-induced fibrogenic reactions were markedly reduced in *caspase 1*- and *Tlr2*-deficient mice, respectively, indicating that the Nlrp3 inflammasome participates in NASH. The group further developed *Nlrp3* knock-in mice. These mice exhibited severe liver inflammation, hepatocyte pyroptosis, stellate cell activation and collagen deposition, which was at least partially suppressed by an Il-1 receptor antagonist.

Edward N Harris (University of Nebraska, Lincoln, NE, USA) gave a talk on hepatic sinusoidal endothelial cells (LSECs) in NAFLD. Rats were fed a high-fat diet (HFD) to generate fatty livers. No accumulation of lipid was observed in LSECs, which exhibited nearly intact fenestration, similar to normal controls. Stabilin 2 mRNA expression was downregulated, explaining the increased level of plasma hyaluronan observed in NAFLD.

Mashani Mohamad (University of Sydney, Sydney, Australia) gave a talk on the defenestration of liver endothelial cells in insulin resistance. F344 rats were treated with poloxamer 407 (P407), a synthetic surfactant that causes the defenestration of LSECs and hyperlipidemia. The multiple indicator dilution method demonstrated that the hepatic volume of distribution as a fraction of the extracellular space was significantly decreased for both insulin and glucose in P407-treated rats. Insulin receptor substrate 1 protein phosphorylation was also diminished in the treated rats. These results identify a role for LSEC structure in insulin resistance.

Kenichi Ikejima (Juntendo University, Tokyo Japan) gave a talk on the effects of dietary glycine on metabolic steatohepatitis in diabetic KK-A^y^ mice. KK-A^y^ mice fed a glycine-enriched diet exhibited limited development of hepatic steatosis and inflammatory infiltration along with reduced expression of hepatic sterol regulatory element-binding protein (Srebp)1c, fatty acid synthase, and acetyl CoA carboxylase. Furthermore, he reported that dietary glycine significantly increased NKT cell fractions and mRNA levels of arginase-1, a marker of M2 macrophage transformation, in KK-A^y^ mice. He concluded that glycine is a promising immune-nutrient for the treatment of NASH.

Jacquelyn J Maher (University of California, San Francisco, San Francisco, CA, USA) gave tutorial talk 7. Alcohol and excess nutrients are cellular stressors that trigger an 'ER stress’ response that contributes to the pathogenesis of steatosis and steatohepatitis in ASH, NASH, and other liver diseases. ER stress generally causes cell death, inflammation, and fibrosis, which are mediated by the activation of JNK and CHOP, the latter of which does not play a central role in the pathogenesis of MCD-induced steatohepatitis. A recent study indicated that ER stress is involved in the stellate cell activation process; thus, ER stress may be a therapeutic target. Expanding upon this idea, she emphasized the utilization of bile acids as chemical chaperones with hepatoprotective properties. Obeticholic acid, a farnesoid X receptor (FXR) agonist structurally similar to chenodeoxycholic acid, has demonstrated positive effects in clinical trials for type 2 diabetes and NAFLD.

Akiko Eguchi (University of California, San Diego, La Jolla, CA, USA) reported the therapeutic efficacy and safety of suppressing liver Bid using RNA interference or a Bid inhibitor. Mice were fed a CDAA diet for 19 weeks to induce NASH. After 3 weeks of treatment with siRNA and Invivofectamine® Rx, Bid mRNA expression was suppressed by 50%, Bid protein levels were reduced to 10%, and a marked improvement in liver fibrosis was observed. Similarly, the administration of a truncated Bid inhibitor for 4 weeks improved liver fibrosis and suppressed the expression of hepatic inflammatory genes such as Il-1β, Il-6, and Tnfα. She concluded that Bid inhibition might be useful as an antifibrotic NASH therapy.

Koichi Miura (Akita University, Akita, Japan) gave a talk on the role of Tlrs and their downstream effector molecules in the development of NASH. A CDAA diet was used to induce NASH in mice. Several lines of gene-deficient mice were used in this study. The data demonstrated that both fibrosis and inflammatory reactions were alleviated in *Tlr2*^*-/-*^, *Tlr4* mutant, *Tlr9*^*-/-*^, and *MyD88*^*-/-*^ mice. In particular, macrophages were considered to contribute to the progression of NASH in this model because they produced cytokines and chemokines and expressed inflammatory molecules such as Nlrp3, Nlrp1, Asc, and Aim2. In addition, the CCR2 inhibitor RS102895, markedly improved NASH.

Tadashi Moro (Tokai University, Isehara, Japan) reported the effects of oxidative stress and high fat intake on hepatic inflammation and fibrogenesis in Tet-mev-1 mice. He demonstrated that the induction of mitochondrial superoxides results in mitochondrial membrane potential in primary hepatocytes and contributes to the activation of the *col1a2* promoter in co-cultured hepatic stellate cells. *In vivo* studies showed that doxycycline-treated Tet-mev-1 mice fed a high fat/high sucrose diet expressed increased C-C chemokine mRNAs, such as chemokine ligand 2 (Ccl2), Ccl3, and Ccl4, without apparent fibrosis. This model can be used to evaluate the direct contribution of mitochondrial oxidative stress to the progression of liver disease.

### Poster session

Forty-four posters were divided into six categories: (1) Hepatic Stellate Cells, four posters; (2) Liver Fibrosis, 11 posters; (3) Cancer Microenvironment, six posters; (4) Inflammation and Immunity, 11 posters; (5) NASH, nine posters; and (6) ASH, three posters. The president selected seven posters from this group, and the authors were invited to speak at the 'Short Oral Presentation of Poster’ session. The presentations included the following: 'Hepatic stellate cell and biodiversity’ by Haruki Senoo (Akita University, Akita, Japan); 'Effect of leptin and ethanol on the regulation of apoptosis and fibrosis in the mouse HCC cell lines’ by Balasubramaniyan Vairappan (Jawaharlal Institute of Postgraduate Medical Education and Research, Pondicherry, India); 'GP130 mediates inflammation-associated hepatocellular carcinogenesis in hepatocyte specific *Tak-1* deleted mice’ by Yoon Seok Noh (University of California, San Diego, La Jolla, CA, USA); 'In vitro liver models: design and cellular interaction studies’ by Srivastan Kidambi (University of Nebraska-Lincoln, Lincoln, NE, USA); 'IFN-*γ* deficiency attenuates hepatic inflammation and fibrosis in mouse steatohepatitis’ by Terumi Takahara (Toyama University, Toyama, Japan); 'Stress accelerates derangement of bile acid homeostasis during development of steatohepatitis in mice’ by Tsutomu Matsubara (Osaka City University, Osaka, Japan); and 'Protein S exacerbates acute alcohol-induced liver injury by enhancing activation of liver natural killer T cells’ by Motoh Iwasa (Mie University, Tsu, Japan).

## Conclusions

As usual, this symposium focused on discussions of the structure and function of hepatic sinusoidal cells, including Kupffer cells, sinusoidal endothelial cells, stellate cells, pit cells, and dendritic cells. However, this Osaka symposium facilitated the concept of 'Bridging Basic Science and Clinical Hepatology’, following the precedent set at the previous meeting in Florence, Italy, 2011. As progress accelerates the growth of hepatological science and medicine, it is necessary to promote the interchange of knowledge between basic and clinical scientists. To this end, topics in this symposium included the involvement of hepatic sinusoidal cells in the pathogenesis of liver fibrosis, inflammation and immunological reactions caused by viral infection, alcohol abuse, and lifestyle-related steatohepatitis, and their applications in the development of therapeutic regimens for treating liver disease. Sinusoidal cell involvement in hepatic carcinogenesis was thoroughly argued. Furthermore, the presentations on the advancement of research on the fibrosis of extrahepatic organs were outstanding.

The 18th ISCHS symposium will be held in Asilomar, California, USA in October 2015. Please check the ISHSR website (http://www.ishsr.net) for further information. See you in Asilomar in 2015!

## Abbreviations

Adh: Alcohol dehydrogenase; APAP: Acetaminophen; ARB: Angiotensin II receptor blocker; ASH: Alcoholic steatohepatitis; BCAA: Branched chain amino acid; BM SPCs: Bone marrow sinusoidal cell progenitor cells; Ccl2: Chemokine ligand 2; CCl4: Carbon tetrachloride; CCr: Chemokine receptor; CDAA: Choline-deficient amino acid-defined; Chd: E-cadherin; Col1a1: Collagen 1a1; Cox-2: Cyclooxygenase 2; DCA: Deoxycholic acid; DEN: Diethylnitrosamine; EMT: Epithelial-mesenchymal transition; ER: Endoplasmic reticulum; FXR: Farnesoid X receptor; GnT-V: N-acetylglucosaminyltransferase V; HCC: Hepatocellular carcinoma; HCV: Hepatitis virus C; HFD: High-fat diet; Hgf: Hepatocyte growth factor; HSRJ: Hepatic Sinusoidal Research Japan; Icam: Intercellular adhesion molecule; Ifn: Interferon; Il: Interleukin; Isg: Interferon-stimulated gene; ISCHS: International Symposium on Cells of the Hepatic Sinusoid; LPS: Lipopolysaccharide; LSECs: Liver sinusoidal endothelial cells; Mcp: Monocyte chemotactic protein; Mmps: Matrix metalloproteinases; NASH: Non-alcoholic steatohepatitis; Nlrp3: Nlr family, pyrin domain containing 3; Nrf2: Nuclear factor erythroid 2-related factor 2; Pdgf: Platelet-derive growth factor; Plgf: Placenta growth factor; Pparγ: Peroxisome proliferator-activated receptor γ; RA: Retinoic acid; SASPs: Senescence-associated secretary phenotypes; SPCs: Sinusoidal cell progenitor cells; Srebp: Sterol regulatory element-binding protein; Tak1: Tgf-β–activated kinase 1; Tgf-β: Transforming growth factor β; Timp: Tissue inhibitor of matrix metalloproteinase; Tlrs: Toll-like receptors; Tnfα: Tumor necrosis factor α; uPA/SCID: Urokinase plasminogen activator/severe combined immunodeficient; VEGFR: Vascular endothelial growth factor receptors.

## Competing interests

The author declares that he has no competing interests.

